# Medial sigmoid depression prevalence and association with a sigmoid notch: cone beam computed tomography and panoramic image study

**DOI:** 10.1038/s41598-024-62490-5

**Published:** 2024-05-21

**Authors:** Ozlem Busra Dogan, Hatice Boyacioglu

**Affiliations:** Department of Dentomaxillofacial Radiology, Faculty of Dentistry, Hacettepe University, 06230 Sihhiye, Ankara, Turkey

**Keywords:** Anatomy, Diagnostic imaging, Mandible, Morphology, Orthognathic surgery, Anatomy, Dentistry, Medical imaging

## Abstract

This study aims to determine whether and how the data of the medial sigmoid depression (MSD) area via cone beam computed tomography (CBCT) differs from panoramic radiography. This study also aims to evaluate various sigmoid notch types and assess the relationship between sigmoid depression and notch morphology. A total of 129 individuals consisting of 258 sides were evaluated. Chi-Square/Fisher Exact tests were used to assess parameters on a categorical scale between two or more groups. McNemar’s test compared the findings detected on panoramic and CBCT images. MSD was more prevalent in females than males in both techniques, but this difference was not statistically significant. There was no association between the prevalence of MSD and the morphology of the sigmoid notch. The incidence of MSD shape was not significantly different between both imaging modalities. In both panoramic and CBCT, we found a high and similar prevalence of MSD. While the MSD prevalence was 66.7% for CBCT, it was 58.1% for panoramic. The shape or prevalence of MSDs in either approach did not correlate with sigmoid notch morphology. The two approaches' identical prevalence indicates that the panoramic image has adequately defines MSD. The high prevalence of MSD demonstrated how important it is for clinicians to characterize this anatomical variation accurately for the surgical treatment.

## Introduction

Sigmoid notch (SN) is a deep concavity on the mandible's upper border encircled by the condyle and coronoid process^1^. The coronoid process and the condyle are divided by the SN, also known as the mandibular notch (MN). It allows blood to travel to and from the masseter muscle via the masseteric artery and vein. The coronoid process and mandibular condyle both have an impact on the shape of SN. Shakya et al. have described morphological variations of the SN (broad-wide, round, and sloping). These variations occur due to the genetic background or functional changes with the growth progression^2^.

The term ‘medial sigmoid depression’ (MSD) was first used in a study on dry human skulls by Langlais et al. in 1983^3^. MSD occurs by reduced radiation absorption due to the bone's thinning on the lingual side of the sigmoid notch^4^. Foramen-like radiolucency can be seen in the upper ramus immediately below and somewhat anterior to the sigmoid notch, indicative of MSD. Bilateral or unilateral radiolucency is conceivable. It can be confused with various pathologies due to its location and appearance. Clark called this depression a pseudocyst in 1984 and examined it in one hundred skulls^5^.

Recently, Nyer Firdoose identified one such variation in the structural morphology of the mandibular coronoid process, which has been elaborated as the coronoid foramen in the oro-facial region^6^. Even though MSD has long been understood to be an anatomical variation, it might be mistaken for an abnormal coronoid foramen when seen on panoramic radiography^6,7^. Several other articles in the literature also mentioned MSD as a possible radiographic finding or a differential diagnosis for other pathologies^8–12^. A dentist may suggest that the patient undergo three-dimensional imaging since MSD can visually resemble a radiolucent lesion. In contrast to panoramic radiography, cone beam computed tomography (CBCT) imaging offers superior image quality and permits precise three-dimensional visualization of anatomic variations like mandibular accessory foramina and minute nutrient canals. This is because real anatomy exists in three dimensions^6^. Although salivary gland-related cortical defects (stafne bone defect) have been reported to occur in more posterior and/or inferior locations in the medial or lateral aspect of the mandibular ramus, Defects involving the ascending ramus have also been reported. Thus, while making an MSD differential diagnosis, these lesions should also be considered^13–15^.

MSD area is thin; therefore, it may increase the potential for complications in orthognathic surgery^16^. While performing orthognathic surgery, particularly sagittal split osteotomies and gross bone resections for mandibular corpus cancers, MSD is crucial in determining the osteotomy locations with the lowest risk of fracture^17^. Furthermore, it has been shown that MSD is linked to high levels of muscular activity, which raises the risk of recurrence after orthognathic surgery^18^. In addition, the fusion of the medial and lateral cortical plates induced by MSD may raise the risk of difficult or undesirable fractures^18^. It needs no additional radiological study or intervention if no surgical treatments are planned at this spot (such as sagittal split osteotomy orthognathic surgery). Surgery complications might be avoided by knowing this anatomical landmark's prevalence and shape variability^19^. Carvalho et al. classified the morphology of MSD into tear-drop, semilunar, circular, and triangular^4^. Most studies investigating MSD have focused on panoramic radiographs, which have several limitations, such as distortions, superpositions, and magnifications. The sigmoid notch region is displayed on the panoramic images along with the nasopharyngeal airway shadow, pterygoid plate, and soft palate. Therefore, the observer may overlook medial sigmoid depression^19^. On the other hand, cone-beam computed tomography (CBCT) devices providing three-dimensional (3D) imaging eliminated these limitations.

MSD is a relatively recently described anatomical variation. Because of this region's fragility, separating the ramus will likely be more challenging^16^. Despite being in a location at risk for surgical complications, there are not many studies that assess this anatomical variation^4,20,21^. Moreover, no studies can be found comparing the MSD on CBCT and panoramic. Whether panoramic imaging, which contains a lower radiation dose than CBCT imaging, is sufficient to detect this structure must be determined. The morphology of anatomical structures is affected by nearby structures. The MSD is just in front of the SN. We could not find a study examining the association between the SN and MSD presence and morphology. This study aimed to assess the prevalence of MSD area and whether the prevalence differs between panoramic and cone beam computed tomography (CBCT) images. Additionally, this study aims to assess different sigmoid notch types and determine whether there is a relevance between sigmoid depression and notch morphology.

## Material and methods

This study was performed retrospectively. The Hacettepe University's Local Ethics Committee approved the experimental procedure (GO 21/705). For the sole purpose of this study, no individuals were exposed to radiation, the included images had been acquired for routine therapies and clinical evaluations in 2021. The CBCT images were obtained using an i-CAT Next Generation CBCT scanner (Imaging Sciences International, Hatfield, PA, USA) with the following settings: 5 mA (tube current), 120 kVp, 14.7–17.8 s (exposure time), 16 × (8–13) cm and 23 × 17 cm (fields of view), and 0.20–0.30 mm (voxel size). All CBCT scans were obtained in a standardized head posture (the Frankfort plane parallel to the floor). The CBCT scans were reconstructed 3-dimensionally so that they could be sectioned at any plane and position. The panoramic images were obtained using a Veraview IC5 HD (Morita Corporation, Osaka, Japan) with the following settings: 12 mA, 18 s, and 70 kV. CBCT and panoramic images were selected from the digital archive of the Department of Dentomaxillofacial Radiology, Faculty of Dentistry, at the University of Hacettepe. The inclusion criteria were individuals older than 18 years, panoramic and CBCT imaging availability, and clear imaging of both full sigmoid notches. The exclusion criteria were: A lesion or tumor in the SN region, or poor imaging quality (asymmetrical magnification, inappropriate patient positioning, and blurring).

Panoramic and CBCT images of 129 individuals met the inclusion criteria. The same observer (maxillo-facial radiologist with 5 years of experience) evaluated anonymous CBCT and panoramic images separately. The same computer and darkroom were used to review every image. The observer could only adjust the brightness and contrast to evaluate. The assessments of the CBCT images were conducted on the CBCT reformatted panoramic screen and axial section. Frankfort Horizontal was utilized to standardize head locations anteroposteriorly in the reconstructed images. Maximal overlap of bilateral components in the mandibular body, ramus, and maxilla was used in sagittal orientation. By choosing a unique focal trough that extended posterior to the condyles and went through the coronoid process, panoramic images were generated from CBCT volumes. Focal trough width was varied to ensure it encompassed the entire length and height of the ramus mandibula. Axial serial slices were reviewed to ensure the focal trough encompassed complete of the ramus mandibula. The right and left sides were assessed.

**Figure 1:**
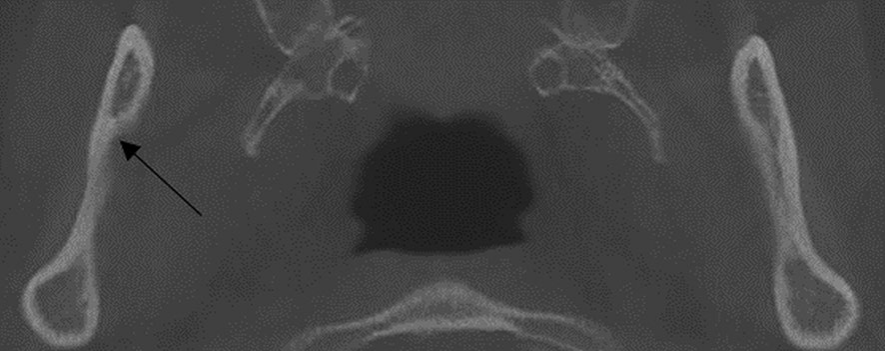
Confirmation of MSD presence in CBCT axial section.

Firstly, the presence of MSD was confirmed in the axial section of the CBCT image then the presence or absence of MSD was recorded on the reformatted panoramic screen (Fig. 1).

**Figure 2:**
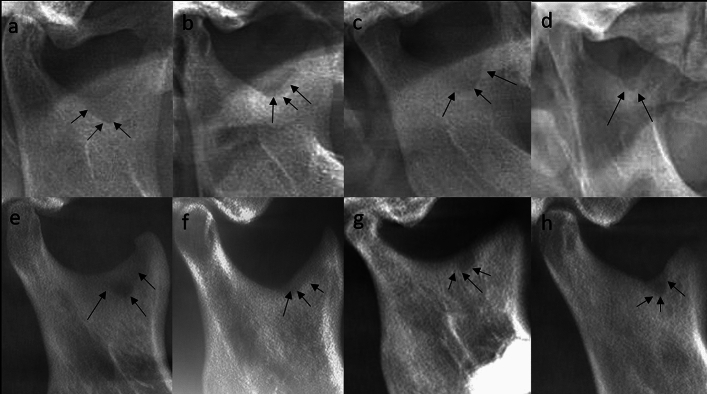
The cropped panoramic image of the MSD (**a** showing tear-drop shape, **b** showing semilunar shape, **c** showing circular shaped, **d** showing triangular shape; The cropped CBCT panoramic image of the MSD (**e** showing tear-drop shape, **f** showing semilunar shape, **g** showing circular shape, **h** showing triangular shape).

Secondly, if it exists, the shape is noted. The literature has characterized the geometric shapes of MSD as tear-drop, semilunar, circular, and triangular, which are the kinds evaluated for interpretation^4^ (Fig. 2).

**Figure 3:**
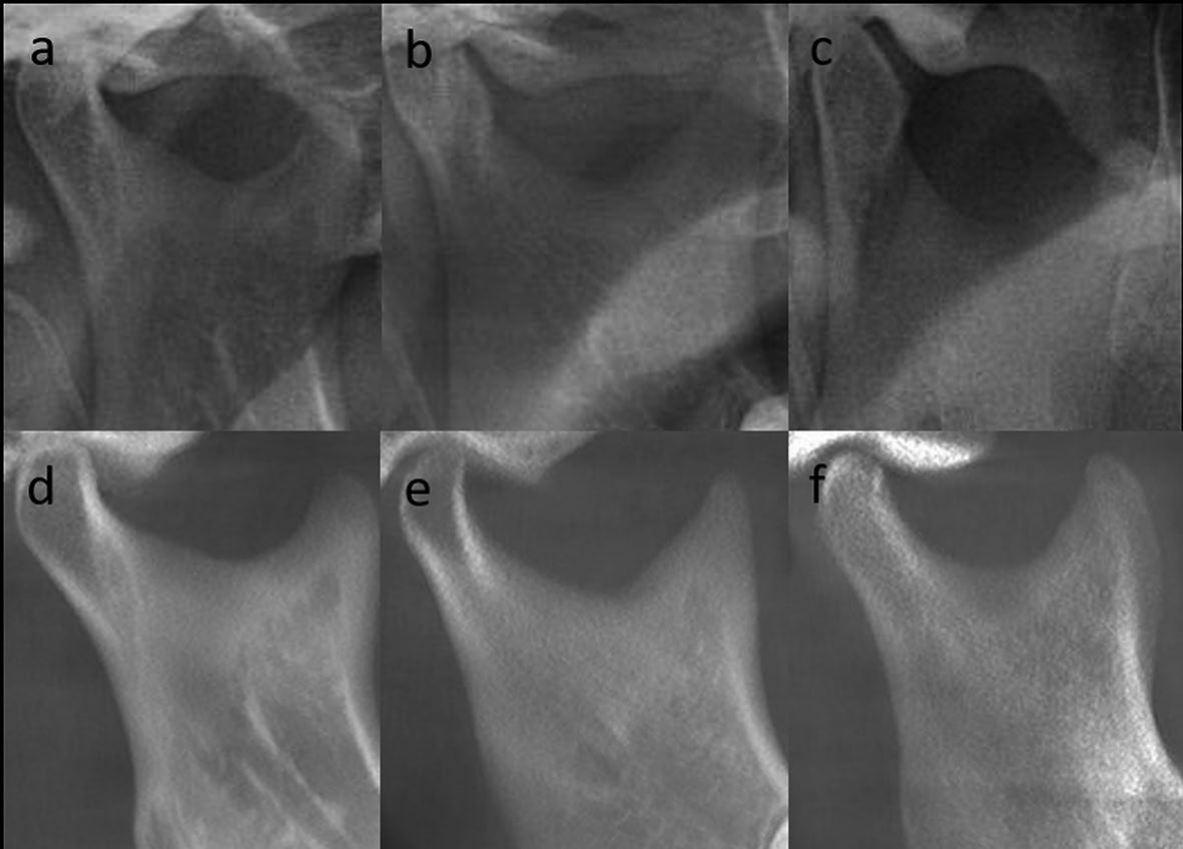
The cropped panoramic image of the SN (**a** showing sloping shape, **b** showing wide shape, **c** showing round shape); Cropped CBCT reformatted panoramic image of the SN (**d** showing sloping shape, **e** showing wide shape, **f** showing round shape).

Thirdly, the sigmoid notch morphology was analyzed by the classification given by Shakya et al.^2^ (Fig. 3) as sloping, wide and round and noted. The aforementioned forms were also noted using panoramic images.

Twenty-five randomly selected CBCT and panoramic images were re-evaluated to explore intra-observer consistency three weeks after the first evaluation.

### Statistical analysis

A non-parametric framework was employed for the analysis of the qualitative data in order to determine the significance of research parameters on a categorical scale between two or more groups using the Chi-Square/Fisher Exact test. The Fisher Exact test was applied to examine the normal distribution of the variables with small cell samples. The significance level for interpreting the data was set at 0.05; when p 0.05, a significant relationship between variables was found. However, when p > 0.05, there was no significant correlation with the variables on a categorical scale. Cohen's kappa coefficient was calculated for intra-observer consistency.

McNemar’s and McNemar Bowker tests compared the findings detected on panoramic and CBCT images, with a significance level of 0.05 (α = 5%). The images were analyzed using SPSS (Statistical Package for Social Studies) version 23.00. The statistical significance was set at p˂ 0.05.

### Ethical approval and ınformed consent

The Local Ethics Committee of Hacettepe University, retrospectively registered, with the number of GO 21/705. All procedures followed were in accordance with the ethical standards of the responsible committee on human experimentation (institutional and national) and with the Helsinki Declaration of 1975, as revised in 2008 (5). Informed consent was obtained from all patients for being included in the study.

## Results

Among 129 individuals consisting of 258 sides, the prevalence and morphology of MSD and the sigmoid notch (SN) morphology were evaluated using CBCT and panoramic images. The sample comprised 48 males and 81 females, aged 18 to 78 years (mean age of 46.6 ± 15.4 years). Except for gender, all data and tables are provided for 258 sides (n = 258). There is no distinction between right and left.

The Kappa test was used for intra-observer variability, and The Kappa value was found between 0.75 and 0.85, showing a near-perfect agreement for CBCT and panoramic images.

The prevalence of sigmoid notch (SN) morphology was as wide at 51.9% (n = 134), followed by a round at 29.1% (n = 75), and sloping at 19% (n = 49) in CBCT. The prevalence of sigmoid notch morphologies was detected as wide in 47.7% (n = 123), followed by a round in 31% (n = 80), and in sloping 21.3% (n = 55) in panoramic.

MSD was more prevalent in females than males in both techniques, but this difference was not statistically significant. Table 1 shows the prevalence of MSD on panoramic and CBCT images with regard to gender.

**Table 1:** The prevalence of MSD on panoramic and CBCT images according to gender.

			Panoramic		Total	CBCT		Total
			Absent	Present		Absent	Present	
Gender	Female	Count	31	51	82	26	56	82
		% within gender	37.8%	62.2%	100.0%	31.7%	68.3%	100.0%
	Male	Count	23	24	47	17	30	47
		% within gender	48.9%	51.1%	100.0%	36.2%	63.8%	100.0%
Total		Count	54	75	129	43	86	129
		% within gender	41.9%	58.1%	100.0%	33.3%	66.7%	100.0%
		n = 129	χ^2^ = 1.52 p = 0.21			χ^2^ = 0.26 p = 0.60		

The absence and presence of MSD on both techniques were insignificant (Mc nemar p = 0.81 Table 2).

**Table 2:** The prevalence of MSD in panoramic and CBCT imaging.

		CBCT		Total
		Absent	Present	
Panoramic	Absent	60	37	97
		61.9%	38.1%	100.0%
	Present	34	127	161
		21.1%	78.9%	100.0%
Total		94	164	258
		36.4%	63.6%	100.0%
n = 258		p = 0.81		

MSD was detected in 63.3% (n = 164) of CBCT images. Triangular was the most prevalent morphology identified in 36% (n = 59) of MSD by semilunar in 34.1% (n = 56), tear-drop in 24.3% (n = 40), and circular in 5.5% (n = 9). MSD was detected in 62.4% (n = 161) of panoramic images. Triangular was the most prevalent morphology identified in 37.2% (n = 60), followed by tear-drop 33.1% (n = 53), semilunar in 24.2% (n = 39), and circular in 5.5% (n = 9). The incidence of MSD shape was not significantly different between both imaging modalities (McNemar Bowker p = 0.07 Table 3).

**Table 3:** The prevalence of MSD shape on panoramic and CBCT imaging.

		CBCT					Total
		Absent	tear-drop	Semilunar	Circular	Triangular	
Panoramic	Absent	60	3	12	1	21	97
		61.9%	3.1%	12.4%	1.0%	21.6%	100.0%
	Tear-drop	12	26	8	0	7	53
		22.6%	49.1%	15.1%	0.0%	13.2%	100.0%
	Semilunar	9	3	21	2	4	39
		23.1%	7.7%	53.8%	5.1%	10.3%	100.0%
	Circular	1	0	5	1	2	9
		11.1%	0.0%	55.6%	11.1%	22.2%	100.0%
	Triangular	12	8	10	5	25	60
		20.0%	13.3%	16.7%	8.3%	41.7%	100.0%
Total		94	40	56	9	59	258
n = 258		36.4%	15.5%	21.7%	3.5%	22.9%	100.0%

There was no association between the shape of MSD and the morphology of the sigmoid notch in both techniques (Table 4 for panoramic imaging, Table 5 for CBCT imaging).

**Table 4:** The relationship between the shape of MSD and the morphology of the sigmoid notch on the panoramic image.

		Sigmoid Notch in Panoramic			Total
		Sloping	Wide	Round	
MSD Shape in Panoramic	Absent	19	45	33	97
		19.6%	46.4%	34.0%	100.0%
	Triangular	14	26	13	53
		26.4%	49.1%	24.5%	100.0%
	Semilunar	7	21	11	39
		17.9%	53.8%	28.2%	100.0%
	Circular	1	5	3	9
		11.1%	55.6%	33.3%	100.0%
	Tear-drop	14	26	20	60
		23.3%	43.3%	33.3%	100.0%
Total		55	123	80	258
n = 258		21.3%	47.7%	31.0%	100.0%

**Table 5:** The relationship between the shape of MSD and the morphology of the sigmoid notch on CBCT.

		Sigmoid Notch shape in CBCT			Total
		Sloping	Wide	Round	
MSD shape in CBCT	Absent	22	44	28	94
		23.4%	46.8%	29.8%	100.0%
	Triangular	12	20	8	40
		30.0%	50.0%	20.0%	100.0%
	Semilunar	4	35	17	56
		7.1%	62.5%	30.4%	100.0%
	Circular	1	5	3	9
		11.1%	55.6%	33.3%	100.0%
	Tear-drop	10	30	19	59
		16.9%	50.8%	32.2%	100.0%
Total		49	134	75	258
n = 258		19.0%	51.9%	29.1%	100.0%

## Discussion

We retrospectively assessed MSD and SN in panoramic and CBCT images in the present study. In order to evaluate the repeatability of the method in CBCT and panoramic images, the intra-observer agreement was evaluated, and the consistency was found to be relatively high in all of the evaluations. The panoramic technique is widely used as a dental imaging modality. Various studies of SN and MSD on panoramic images have evaluated the relationship between demographic and etiologic factors^2,20^.

Developmentally, the coronoid process, mandibular condylar process, and mandibular notch configurations are interconnected^22^. The mandibular notch formation is highly reliant on the coronoid and condylar processes contours^23^. Nevertheless, genetics, hormones, eating habits, and temporalis muscle activity may all affect how these structures shape morphologically. In the literature, there have been previous researches in which various configurations of these structures have been demonstrated^23–25^. According to the study Shakya et al., SN was classified into the following types: wide, round, and sloping^2^. Similar to Sahithi et al. and Manoj et al.'s studies, our study observed the most common type of wide SN on panoramic images^23,25^. By contrast, Shakya et al. and Dar et al., reported sloping SN as the most frequent type^2,26^.

In our study, the most common type of wide SN was observed on CBCT, similar to panoramic images. By contrast, Tassoker et al. reported round SN as the most frequent type^27^. Nevertheless, different sections were evaluated in two studies. Unlike our study, we could not find a study evaluating sigmoid notch in the reformat panoramic screen of CBCT.

The association between MSD and SN morphology was evaluated in our study. No previous research has investigated the association between SN and MSD. No statistically significant relationship was found in either panoramic images or CBCT.

The present study's 62.4% prevalence of MSD in panoramic images was higher than the associated findings from Langlais et al. (10%), Carvalho et al. (20.3%), Kumar et al. (23.2%), and Kang et al. (33%)^3,4,19,28^. Like us, Asdullah et al. reported a prevalence of 70% for MSD in panoramic^20^. Varying techniques and the subjectivity of radiographic interpretation might explain these discrepancies. MSD may not be seen due to positioning errors^20^. According to Langlais et al., when the MSD is absent, it may occasionally be because the location is not in the focal trough^3^.

According to the study Carvalho et al., MSD was classified into the following types: tear-drop, semilunar, circular, and triangular. Similar to Carvalho et al.’s study, triangular MSD was the type most frequently seen, whereas circular shape was the least frequently seen on panoramic images^4^. By contrast, Asdullah et al. reported semilunar, the type most often^20^.

Storey et al. have suggested that the size and shape of the depression may be a result of variations in muscle function^29^. The functional adaptation in the ramus caused by the insertion of the medial and posterior attachments of the temporal muscle into this region plays a significant role in characterizing the features of MSD. Adisen et al. found higher maximum biting force values in MSD patients, demonstrating a correlation between MSD and maximum bite force^18^. We could not evaluate the subjective factors affecting muscle activity since our investigation was retrospective.

MSD can be confused with various pathologies due to its radiolucent appearance. As a potential radiography finding or as a differential diagnostic for other conditions, MSD was mentioned in passing in several additional literature articles^8–12^. Although rare, studies are reporting that stafne bone cyst is detected in the ascending ramus. It has been reported that MSD may be confused with a stafne bone defect. However, these conditions can be easily distinguished on panoramic radiographs^13–15^.

Considering that MSD can mimic a nonodontogenic cystic lesion in appearance, a dentist may refer the patient to undergo three-dimensional imaging. CBCT yields three-dimensional (3D) images, eliminating limitations, and thus has attracted increasing interest. CBCT-reformatted panoramic images used in the current study were free of magnification, superimposition of surrounding structures, and other issues endemic to panoramic radiography since they were reformatted slices of the maxilla and mandible^30^. CBCT equipment is easily accessible in many dental clinics. For this reason, the possibilities of acquiring CBCT images from the regions that clinicians consider suspicious have become considerably easier. Nonetheless, it is essential to carefully consider if the patient's request for an advanced examination is necessary and whether it is preferable to alternative methods.

MSD can be misinterpreted as a coronoid foramen. Even after a CBCT examination MSD may still be misinterpreted as a foramen because of inappropriate thresholding settings in transparent volume-rendered images, or it may be misinterpreted as a break in the bone surface in computed tomography (CT) sections because of metal artifact from metallic objects in the oral cavity, such as dental prosthetic components or filling materials^6,7^. The threshold, opacity, and translucency settings in volume-rendered (VR) CT images were selected to display structures deep within the bone surface; consequently, MSD can be erroneously interpreted as a foramen since very thin bone is not visible in such VR images because of the thresholding process^13,31^. The detection of pseudo foramina in VR CBCT images has been classified by the European Commission Guidelines on CBCT as a post-acquisition manipulation error because of improper thresholding, and operator training in the proper application of windowing controls is advised^32^. Multiplanar reformatting (MPR) images provide the most accurate results when evaluating thin bone, with measurements that have the highest agreement with anatomical findings^33^. Volumetric reformatting of CT has been shown to be less accurate than MPR, and it should therefore be borne in mind that such reformatting of CT or CBCT examinations is for general visualization purposes only and not for diagnosis and analysis. For this reason, volumetric reformatted images were not used in our study^13,34^.

The present study's prevalence of MSD was 63.6% in CBCT images. We found that Hasani et al. was the only author to analyze the prevalence and morphology of depression on CBCT images. Like us, Hasani et al. reported a prevalence of 60.45% for MSD^21^. In our study, triangular MSD was most frequently observed, while circular was less frequently observed in CBCT. By contrast, Hasani et al. reported semilunar, the type most often. Hasani et al. assessed the reconstructed 3D images, while we assessed the reformatted panoramic images.

We evaluated whether CBCT imaging was needed to detect MSD. The prevalence of MSD in CBCT and panoramic images showed remarkably similar results in our investigation. Although we found that MSD prevalence was high and similar in panoramic and CBCT images, this finding was insignificant. The presence of MSD identified in CBCT images was sometimes not detected in panoramic images or MSD detected in panoramic imaging could not be confirmed in CBCT images, which is the gold standard for identification. The differences in the results may be related to the subjectivity of radiographic interpretation of the morphology due to the panoramic image techniques limitations and the observer's skill. It is important to evaluate intraobserver and interobserver agreement in radiographic studies. Although intraobserver agreement was found to be high in our study, the fact that interobserver agreement was not evaluated is one of the limitations of the study. MSD In our study, triangular MSD was the most frequently seen, whereas circular shape was the least frequently seen on panoramic and CBCT images. By contrast, the prevalence of tear-drop and semilunar shapes of MSD differed markedly in panoramic and CBCT images. The MSD shape was quite difficult to identify both on panoramic images and on CBCT images. These results can be impacted by the expert's subjectivity and experience in interpreting the radiograph to define the shape.

In addition, ethnic differences, variances in the device used, discrepancies in the method used to analyze MSD, and variations in sample size may all contribute to the disagreements in the results.

The similar prevalences in the two methods resulted in the conclusion that advanced imaging is not necessary to identify MSD. However, CBCT imaging may be necessary to obtain more information about the MSD morphology for surgical procedures.

## Conclusion

According to our results, the most common SN shape was wide observed in panoramic and CBCT images. We found the prevalence of MSD to be high and similar in panoramic and CBCT. There was no correlation between SN morphology and MSD shape or prevalence in both techniques. In our study, triangular MSD was the most frequently observed, while circular was less frequently observed in CBCT and panoramic. We consider that the similar prevalence of the two methods reveals that the panoramic image has been sufficient to define MSD. The high prevalence of MSD showed that it is essential for clinicians to define this anatomical variation well. If surgery is needed, patients with MSD could need further investigation with CBCT.

## Data Availability

The data that support the findings of this study are available from the corresponding author upon reasonable request.
